# Reply to: Resource-rich Intensive Care Units Improve Health―What Is Next?

**DOI:** 10.31662/jmaj.2022-0011

**Published:** 2022-02-28

**Authors:** Hiroyuki Ohbe, Yusuke Sasabuchi, Hiroki Matsui, Kiyohide Fushimi, Hideo Yasunaga

**Affiliations:** 1 Department of Clinical Epidemiology and Health Economics, School of Public Health, The University of Tokyo, Tokyo, Japan; 2 Data Science Center, Jichi Medical University, Shimotsuke, Japan; 3 Department of Health Policy and Informatics, Tokyo Medical and Dental University Graduate School of Medicine, Tokyo, Japan

**Keywords:** intensive care unit, intensivist, administrative database

We thank Dr. Shime for reading and providing valuable comments on our study in which we investigated the association between resource-rich intensive care units (ICUs) and patient mortality in a Japanese nationwide database ^(1)^. The results obtained showed that care in resource-rich ICUs, characterized by intensivist staffing, a larger ICU area, and 24-hour medical engineer staffing, was associated with lower ICU mortality compared with care in standard ICUs. Here, we are providing responses to address your comments.

In the statistical model, we adjusted for the patient-level characteristics and ICU- and hospital-level characteristics, including annual ICU admission, number of hospital beds, number of ICU beds, teaching hospital, academic hospital, number of dedicated nurses per ICU bed, dedicated pharmacist staffing, and dedicated physical therapist staffing. Therefore, the adjusted results showed that care in the resource-rich ICUs was associated with lower ICU mortality independent of patient volume, the number of hospital and ICU beds, academic hospital, and multidisciplinary healthcare providers.

The question of whether resource-rich ICUs should be spread throughout the country or whether patients with critical illnesses should be centralized in more resource-rich ICUs is crucial. As aforementioned, our study showed that the structure of resource-rich ICUs improved prognosis independently of patient volume. Therefore, spreading the resource-rich ICU and centralizing patients are not mutually exclusive in improving patient outcomes. In fact, the current definition of standard ICUs in Japan does not meet the world’s standard of ICU structure due to the lack of involvement by intensivists ^(2)^. Since previous studies have consistently shown intensivist staffing to be associated with lower mortality ^(3), (4), (5)^, spreading resource-rich ICUs throughout Japan could be an evidence-based policy. We believe that our study provided important evidence to guide policymakers in modifying ICU systems in Japan.

## Article Information

### Conflicts of Interest

None

### Author Contributions

All authors wrote and revised the paper, and HO had primary responsibility for the final content. All authors read and approved the final manuscript.
